# Supplementary material for: Factors influencing career preference in mental health among nursing students and intern nurses in Dar es Salaam, Tanzania
Source: PLOS Glob Public Health. 2023 Jul 14;3(7):e0002108. doi: 10.1371/journal.pgph.0002108 (PMC10348593; doi:10.1371/journal.pgph.0002108)
Supplement: Attachment — Submitted filename: Response to Reviewers Comments.docx [file pgph.0002108.s001.docx]

**Factors influencing career preference in mental health among nursing students and intern nurses in Dar es Salaam, Tanzania**

**Response to reviewers’ comments**

We thank the reviewers for the comments they have provided to improve this study. We have done an extensive manuscript revision with our point-to-point responses to reviewers' comments provided in the table below. Responses provided can be traced in the manuscript's main document labeled ‘manuscript with trach changes. If any of our responses fail to address your comments, please let us know so that we can provide further details.

| **Reviewers Comments** | **Authors Response** |
| --- | --- |
|  |  |
| **Journal Requirements** |  |
| 1. Please amend your detailed Financial Disclosure statement. This is published with the article. It must therefore be completed in full sentences and contain the exact wording you wish to be published.  a. State the initials, alongside each funding source, of each author to receive each grant.  b. State what role the funders took in the study. If the funders had no role in your study, please state: “The funders had no role in study design, data collection and analysis, decision to publish, or preparation of the manuscript.”  c. If any authors received a salary from any of your funders, please state which authors and which funders. | We agree with the comment. A detailed financial disclosure statement has been provided. The initials of the author who received the grant and the role the funders took in the study have been stated. |
| **Reviewer # 1** |  |
| Reviewer #1: Dear Authors, the manuscript is moderately interesting, needs major revisions. Following are some suggestions. | We appreciated the comment |
| Please use Mesh Terms as keywords. | We agree with the comment. Mesh terms have been added as keywords |
| Please enter the period of the study in the abstract. | We accept the comment. The period of the study has been entered in the abstract |
| Please describe the sampling better. | We agree with the comment. The statement that describes the sampling better has been added in the abstract |
| Please in methods create the statistical analysis section better describing the analyzes carried out. | We agree with the comment. We have created the statistical analysis section that better describes the analyzes carried out. |
| Please check the formatting of the tables and the values inside. | We have checked the formatting of tables and the values inside. We have included the ‘No’ response column to the Chi-square Nursing students’ and intern nurses’ career preference in mental health table for |
| Please for logistic regression models show the pseudo-R2 (Pseudo-R-squared). | We agree with the comment. We have included the pseudo-R2 (Pseudo-R-squared) for logistic regression models, specifically, Nagelkerke’s R squared |
| Please in discussion better compare the results obtained with those present in the literature, the bibliography must be expanded. | We agree with the comment. We have tried to compare the results obtained with those present in the literature, and we have expanded the bibliography |
| Please discuss in more detail the implications this study could have for public health, possibly create discussion in subchapter. | We agree with the comment. The implications of our study for public health has been included in the discussion as a separate paragraph |
| Please improve the conclusions, right now they are quite trivial. Talk about how this study can be useful for the political decision-maker to evaluate the situation and possibly what corrective actions could be useful. | We agree with the comment. We have improved the conclusions and included the role the Ministry of Health, higher learning intuitions, and health facilities can play in addressing the challenge of low career interest in mental health. |
| **Reviewer # 2** |  |
| Thanks for the opportunity to review this paper; generally, the paper is well written, with a few recommendations for improvement. | We appreciate the encouraging comment |
| **Abstract** |  |
| Indicate the sample type.  Do you mean recruited to complete the study questionnaires?  mentioned the instrument names.  AOR, adjusted odds ratio. Mention it completely for the first time.  Add an additional recommendation strategy. | We agree with the comment.  The sample type has been mentioned.  We have replaced the term ‘recruited’ with ‘participated’.  The instrument used has been explained.  Long form of AOR has been mentioned completely for the first time.  Additional recommendation strategy has been added |
| **Introduction** |  |
| Recommend addressing and adding paragraphs about career choices, preferences, and influencing factors. | A paragraph addressing career choices/preferences and influencing factors. |
| Try to support your introduction with a conceptual or theoretical framework.  and support your problem statement with previous or related studies. | We agree with the comment. We have included a conceptual framework to support the introduction. We have also added previous studies to support our problem statement. |
| There is a difference between your title and your purpose (career preference or career development), and both are different, so please be consistent. | We appreciate the observation. We have corrected the discrepancy between our title and our purpose |
| **Methods** |  |
| Please indicate sociodemographic characteristics tool items how many items the Career Preference in Mental Health and Related Factors tool has and how it scores them | We agree with the comment.  Items included in the sociodemographic characteristics, and how the Career Preference in Mental Health and Related Factors tool were scored have been described. |
| Add recommendations for education, practice, and research. | We agree with the comment. Recommendations for education, practice, and research have been added |
