# Supplementary material for: Factors influencing career preference in mental health among nursing students and intern nurses in Dar es Salaam, Tanzania
Source: PLOS Glob Public Health. 2023 Jul 14;3(7):e0002108. doi: 10.1371/journal.pgph.0002108 (PMC10348593; doi:10.1371/journal.pgph.0002108)
Supplement: Attachment — Submitted filename: Response to Reviewers Comments.docx [file pgph.0002108.s002.docx]

**Factors influencing career preference in mental health among nursing students and intern nurses in Dar es Salaam, Tanzania**

**Response to reviewers’ comments**

We thank the reviewers for the comments they have provided to improve this study. We have done manuscript revisions with our point-to-point responses to reviewers' comments provided in the table below. Changes made can be traced in the manuscript's main document labeled ‘Revised Manuscript with Trach Changes. If any of our responses fail to address your comments, please let us know so that we can provide further details.

| **Reviewers Comments** | **Authors Response** |
| --- | --- |
| **Reviewer # 1** |  |
| The authors have implemented the manuscript, it now needs minor revisions | We appreciated the comment |
| Please check English and make the text more fluent. | We accept the comment. The English language used has been revised to make the text more fluent |
| Please insert within the limits of the study the discourse that the proposed models do not represent reality in an optimal way, in fact the R-squared is rather low. | We accept the comment. We have included in the limitations of the study the statement that the models used in the multivariate analysis may not represent reality in an optimal way since the R-squared was low |
